# Analysis of 14 Patients With Congenital Nephrotic Syndrome

**DOI:** 10.3389/fped.2019.00341

**Published:** 2019-08-13

**Authors:** Yan Chen, Yanqin Zhang, Fang Wang, Hongwen Zhang, Xuhui Zhong, Huijie Xiao, Yong Yao, Yi Jiang, Jie Ding, Xinlin Hou

**Affiliations:** Department of Pediatrics, Peking University First Hospital, Beijing, China

**Keywords:** congenital nephrotic syndrome, genetic defect, cytomegalovirus infection, clinical manifestation, prognosis

## Abstract

From January 1995 to June 2018, 14 patients with congenital nephrotic syndrome (CNS) were diagnosed in the Department of Pediatrics, Peking University First Hospital. The clinical data were retrospectively studied. Eight patients underwent genetic testing; 7 of them had *NPHS1* mutations (primary CNS), and 1 did not have a mutation. Of the 7 patients with *NPHS1* mutations, 6 died, and 1 had proteinuria. Of the 14 patients, 8 had cytomegalovirus (CMV) infection, and anti-CMV therapy was administered to 7 of them. The other patient was hospitalized in critically ill condition and died before anti-CMV therapy administration. Of the 7 patients who were administered anti-CMV therapy, proteinuria disappeared in 2 patients; 2 patients died; 2 patients were lost to follow up; and 1 patient still had 3+ proteinuria. Three patients had both *NPHS1* mutations and CMV infection. After anti-CMV therapy, proteinuria was resolved in 1 patient but relapsed to 3+ proteinuria due to a new infection. The other 2 patients died. Of 14 patients, only 1 patient underwent renal biopsy, with results showing mesangial proliferative glomerulonephritis pathology, negative CMV inclusion body, and CMV-DNA. In this study, genetic defect could play a primary role in CNS, and CMV could play a secondary role. Primary CNS with *NPHS1* mutations has a poor prognosis. Primary CNS might be accompanied by CMV infection that responds poorly to antiviral treatment. Secondary CNS caused by CMV infection may be cured with antiviral therapy. However, genetic analysis is necessary to exclude genetic defects.

## Introduction

Congenital nephrotic syndrome (CNS) is a rare clinical syndrome that occurs within the first 3 months of life. The clinical manifestations include proteinuria, hypoalbuminemia, edema, and hyperlipidemia. CNS can be classified into two types: primary (hereditary) and secondary (non-hereditary) (1). Primary CNS is usually related to gene mutations that alter the glomerular filtration barrier. The most common type of primary CNS is the Finnish type (*NPHS1* gene mutation). Secondary CNS is usually associated with various types of congenital infections, such as cytomegalovirus (CMV), syphilis, toxoplasmosis, rubella, hepatitis B virus (HBV), and human immunodeficiency virus (HIV) infections. Maternal systemic lupus erythematosus (SLE), maternal steroid/chlorpheniramine use, and neonatal autoantibodies against neutral endopeptidase can also cause secondary CNS (2).

However, only a few cases of CNS have been reported in China, and secondary CNS due to viral infection is not well defined. The prognosis of primary CNS is poor, whereas that of secondary CNS is relatively good when the treatment of secondary causes is available and effective. However, distinguishing between the two CNS types is difficult. CMV infection has been repeatedly reported as the most common etiology for secondary CNS, but evidence of this causality has been unclear.

Here, we describe 14 infants with CNS who were hospitalized in the Pediatric Department of the First hospital Peking University from 1995 to 2018, 8 of whom developed CMV infection. The clinical characteristics, treatment, prognosis, and effect of CMV infection in CNS are summarized.

## Patients and Methods

### Ethical Statement

This study was approved by the Peking University First Hospital Ethics Committee. Written informed consent (for the publication of the cases reported and any potentially identifying information) was obtained from all participants or their parents. All data were analyzed anonymously.

### Patients

From January 1995 to June 2018, 14 patients (8 boys and 6 girls) were diagnosed with CNS at the Department of Pediatrics, Peking University First Hospital. The onset of the symptoms of all patients occurred within the first 3 months of life.

The patients were included based on the following diagnostic criteria for CNS:

1. Proteinuria: qualitative examination ≥ +++, quantitative ≥50 mg/kg/day, urine protein/creatinine (random) >2.0 mg/mg;
2. Hypoalbuminemia: serum albumin <30 g/L;
3. Hypercholesterolemia: total cholesterol >5.72 mmol/L (220 mg/day l);
4. Edema;
5. Onset age <3 months.
  Criteria 1, 2, and 5 are prerequisites (1–3).

### Clinical Data

Clinical data, including sex, age of onset, amniotic fluid volume, and size of placenta were collected. The medical histories of CMV, syphilis, toxoplasmosis, rubella virus, HBV, and HIV infection during pregnancy were also collected.

In addition, information regarding medication use during pregnancy (including steroid use), suspected mercury poisoning, family medical history (especially family members with kidney disease, unexplained edema, and death), and pet exposure was obtained.

### Laboratory Tests

The following three panels of laboratory tests were performed:

1. To confirm the diagnosis of CNS: urine routine test (sterile urine bag or catheterization sampling), 24-h urinary protein (urethral catheterization sampling) or urine protein/creatinine (sterile urine bag or catheterization sampling), serum albumin, and serum cholesterol;
2. To determine the structure and function of the kidney: renal ultrasound; renal function (serum creatinine and creatinine clearance), early kidney injury biomarkers, and urine protein electrophoresis;
3. To explore the possible pathogens: serum CMV IgM and IgG, toxoplasmosis, and rubella virus were examined in 14 cases. HBV, hepatitis C virus (HCV), herpes simplex virus, syphilis, and HIV were also examined. serum and urine CMV-DNA levels were examined in 14 cases.

### Gene Analysis

Informed consent was obtained from the parents of the patients for genetic analysis. The *NPHS1, NPHS2, WT1, PLCEI*, and *LAMB2* genes, which have been previously reported to cause CNS, were analyzed from 1998 to 2014. Genomic DNA was extracted from the peripheral blood lymphocytes of both patients and their parents using the TIANamp Blood DNA Kit (Tiangen Biotech, China). Sanger sequencing was applied. Positive polymerase chain reaction (PCR) products (with specific expected bands) were sequenced with technical help from MyGenostics Co. Ltd. Abnormal results were re-amplified and sequenced, and the parents' DNA samples were also examined. From 2015 to present, the second-generation sequencing method is used for genetic PANEL testing of hereditary kidney disease.

The judging criteria for pathogenicity of unreported variation is family separation analysis (the number of patients who received parental and/or sibling DNA and analyzed).

### Renal Biopsy Pathology

Renal biopsy was performed upon parents' agreement. Histopathological changes were analyzed. CMV inclusion bodies in the kidney tissue were examined using immunohistochemical methods at the Capital Institute of Pediatrics Medical University. Kidney tissue slices were also examined for CMV-DNA by using molecular probes at the Department of Pathology, Peking University Health Science Center. All methods were performed in accordance with the relevant guidelines and regulations.

## Results

### Clinical Features

From January 1995 to June 2018, 2,583 patients were diagnosed with nephrotic syndrome in the Department of Pediatrics, Peking University First Hospital. Of these patients, only 14 had confirmed CNS. Of the 14 patients, 8 were boys, and 6 were girls. The gestational age (GA) was between 35 + 6 weeks and 42 weeks, and the mean GA was 38+1 weeks. The birth weight (BW) ranged from 1,860 to 4,000 g, with a mean of 2,987 g. The onset age was between 1 day and 3 months of life, and the mean was 27.4 days. The mean onset age for patients with Finnish type CNS was 8.4 days. The main symptom for 12 patients was edema. Two patients (cases 6 and 11) had no obvious symptoms except for proteinuria in laboratory tests (Table 1). Three patients (cases 2, 5, and 7) had hypertension. Three patients (cases 1, 8, and 12) were delivered with placentas >800 g, and 2 patients (cases 1 and 12) had less amniotic fluid (amniotic fluid index, AFI <5) (4). All mothers denied a history of SLE. No family histories of unexplained death were found. No families had pets or evidence of mercury poisoning. The families also did not show evidence of syphilis or HBV infection.

**Table 1:** Onset conditions of 14 patients.

**No**.	**Sex**	**Age of onset**	**Chief complaint**	**Plasma albumin level** **(40–55 g/L)**	**Urinary protein**	**Cholesterol** **(3.4–5.2 mmol/L)**
1	M	27 days	Edema	8.4	4+	9.65
2	F	2 months 27 days	Ascites	10.3	3+	–
3	M	2 days	Edema	9.9	4+	7.71
4	F	20 days	Ascites	12.8	4+	8.18
5	F	2 months 24 days	Edema	21.7	3+	10.54
6	M	15 days	Asymptomatic	21.7	3+	3.33
7	M	3 months	Edema	11.9	4+	10.02
8	M	19 days	Edema	9.4	4+	5.82
9	M	16 days	Edema	16.6	3+	7.99
10	M	13 days	Edema	16.6	3+	6.26
11	F	1 days	Asymptomatic	28.4	2+	6.02
12	M	1 days	Edema	11.6	4+	5.09
13	F	2 days	Edema	14.2	3+	7.83
14	F	7 days	Edema	14	4+	7.61

*Case 14 is an abandoned baby*.

All children were delivered uneventfully.

Family history: All 14 families were non-consanguineous. Only 1 patient (case 11) had a family history of renal disease. Eight individuals in the family had proteinuria among three generations. The mother of patient 11 had proteinuria and hypoalbuminemia at 8 months of age. The mother did not undergo renal biopsy or any treatment. The cousin of the mother had proteinuria at 1 year of age and died at 12 years of age.

### Laboratory Test Results

Of the 14 patients, seven patients' parents agreed to indwell urethral catheterization and we got the urine protein of 24 h, seven patients had random urine collected by sterile urine bags. All the patients met the diagnostic criteria of the nephrotic syndrome.

Of the 14 patients, only 1 patient (case 5) had elevated serum creatinine (91 μmol/L; normal range, 30–40 μmol/L) and blood urea nitrogen (BUN) levels (10.6 mmol/L; normal range, 1.8–7.1 mmol/L).

All patients underwent renal ultrasound examination. In case 3, the results revealed renal pelvis separation with an anteroposterior diameter of 1.04 cm. However, this is not a characteristic manifestation of CNS. This may be the process of kidney development. The kidney sizes and structures in the other children were normal.

CMV infection: Of the 14 patients, 8 patients had evidence of CMV infection. Seven patients had active CMV infection (CMV-IgM positive and/or serum CMV-DNA positive). One patient (case 2) had positive urinary CMV-DNA results. He was hospitalized in critically ill condition and died 2 days after admission before antivirus therapy was initiated.

Pathogenic test results for syphilis, HIV, HBV, HCV, toxoplasma, rubella, and herpes simplex virus were all negative.

### Gene Analysis Results

Eight patients underwent nephropathy-related genetic testing. Seven patients had *NPHS1* gene mutations, and 1 (case 6) had both *NPHS1* and *COL4A5* (the gene that causes Alport syndrome) mutations. One patient (case 11) had negative genetic test results. Three patients (cases 3, 6, and 8) had both *NPHS1* gene mutations and CMV infection (Table 2). Other patients did not undergo such testing due to the undeveloped medical condition at the time the testing was offered or lack of consent from their parents. Analysis of variant sites of *NPHS1* gene is shown in Table 3.

**Table 2:** Clinical features of 14 patients.

**No**.	**Plasma creatinine level μmol/L**	**eGFR^a^**	**Gene type**	**CMV infection**	**Proteinuria at present**	**Outcomes**
1	21	106.7	-	Y	2+	-
2	47.3	43.9	-	Y		Death (3 months, 12 days)
3	12	166.7	*NPHS1*	Y		Death (10 months)
4	16	113.7	-	Y	Negative	9 years
5	91	26.4	-	Y	4+	-
6	39	55.4	*NPHS1 COL4A5*	Y	3+	13 months
7	38	70.5	-	Y	Negative	9 years
8	17	115.3	*NPHS1*	Y		Death (2 months)
9	21.7	92.2	-	N	3+	-
10	17.4	108.1	*NPHS1*	N		Death (2 months)
11	53	36.2	Negative	N	3+	5 years
12	14.4	129.2	*NPHS1*	N	4+	Death (3 months)
13	16	120	*NPHS1*	N	4+	Death (4 months)
14	11.2	196.4	*NPHS1*	N	4+	Death (4 months)

*Y indicates patients with CMV infection, N indicates patients without CMV infection, - indicates that the test was not completed*.

a *Schwartz's formula: eGFR = k × height (cm)/plasma creatinine*.

*Case 14 is an abandoned baby. Cases 4 and 7: follow-up in December 2016; case 6: follow-up in April 2016; case 11: follow-up in September 2015; cases 2, 3, 8, 10, 12, 13, and 14: follow-up in June 2018; cases 1, 5, and 9: lost contact*.

**Table 3:** Variant locus analysis in patients with *NPHS1* mutation.

**No**.	**Variation**	**Amino acid change**	**Mutation status**	**Mutation type**
3	c.2663G>A c.3286+5G>A	p. Arg888Lys -	Het Het	Missense
6	c.2396G>T c.1339G>A	p. Gly799Val p. Glu447Lys	Het Het	Missense Missense
8	c.3027C>G c.3478C>T	p. Tyr1009^*^ p. Ary1160^*^	Het Het	Nonsense Nonsense
10	c.1740G>T c.2042G>A	p. Trp580Cys p. Trp681^*^	Het Het	Missense Nonsense
12	c.713-1G>C c.1760T>G	- p. Leu587Arg	Het Het	Missense
13	c.2506+5G>T c.1135C>T	- p. Arg379Trp	Het Het	Missense
14	c.313G>A c.2386G>C	p. Asp105Asn p. Gly796Arg	Het Het	Missense Missense

* Mutations detected in NPHS1 gene.

*Case 14 is an abandoned baby, and no parental verification was performed*.

### Renal Pathology Results

**Figure 1:**
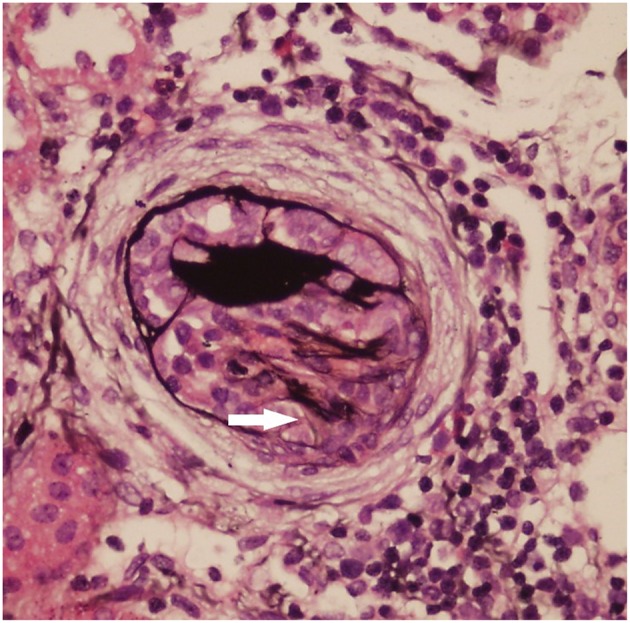
Light microscopy (LM) of the kidney biopsy specimen with periodic acid–silver methenamine (PASM) staining. The mesangial cells and matrix had mild diffuse hyperplasia. One cellular crescent formation can be seen (white arrow).

**Figure 2:**
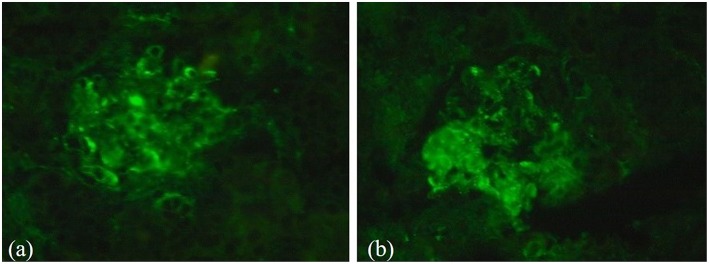
Results of immunofluorescence analysis during renal biopsy. **(a)** Immunofluorescence demonstrated mesangial deposits of C3 (C3+); **(b)** Mesangial deposits of IgM (IgM ++).

**Figure 3:**
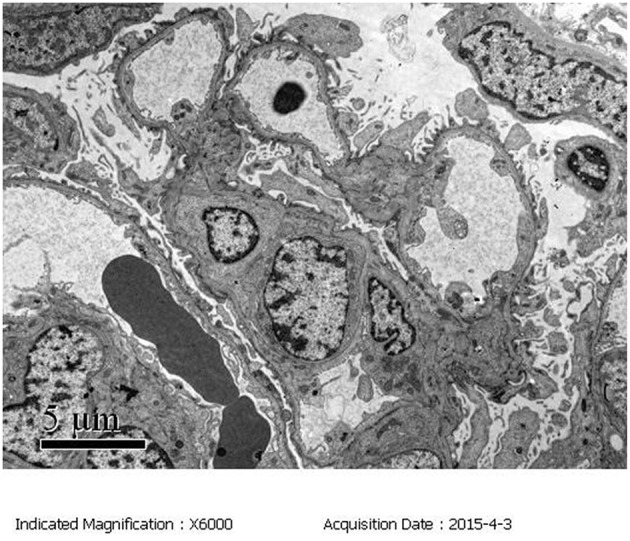
Electron microscopy (EM) of the kidney biopsy specimen. The mesangial cells and matrix exhibited mild hyperplasia. The epithelial foot processes were fused with no electron dense deposits.

Only 1 patient (case 6) underwent renal biopsy at the age of 2 months, and the findings were as follows:

1. Light microscopy: The mesangial cells and matrix showed mildly diffused hyperplasia. Multiple podocytes and immature glomeruli could be seen with cellular crescent formation. Vacuolar and granular degeneration were found in the tubular epithelia. Small focal atrophy, focal lymphoid interstitial mononuclear cells, and eosinophil infiltration with fibrosis could be seen along with thickening of the small artery walls. These changes represented mild mesangial proliferative glomerulonephritis disease (Figure 1).
2. Immunofluorescence demonstrated mesangial deposits of IgM and C3 (IgM ++, C3 +) (Figure 2).
3. Electron microscopy confirmed the light microscopy findings: The mesangial cells and matrix showed mild hyperplasia. The glomerular basement membrane was normal. The epithelial foot process fused together with no electron dense deposits. The tubular epithelia had degenerated, and the lysosomes were increased. The renal interstitium had no obvious lesions (Figure 3).

Renal biopsy sections: immunohistochemistry (IHC): CMV inclusion bodies (-); Molecular Probes: CMV-DNA (-).

The other patients did not undergo renal biopsy due to a lack of consent from their parents, or because their condition was critical after admission to our hospital.

### Treatment

#### Anti-CMV Therapy

In 8 patients with CMV infection, 1 patient (case 2) was hospitalized in critically ill condition and died 2 days after admission.

The other 7 patients were administered ganciclovir as antiviral therapy (5 mg/kg, given twice daily, 12 h apart for 4 weeks). We did not observe any side effects related to ganciclovir therapy.

The urinary protein results became negative after antiviral therapy in 2 patients (cases 4 and 7) and have remained negative up to the follow-up.

Two patients (cases 3 and 8) had *NPHS1* gene mutations combined with CMV infection. After the antiviral therapy, the clinical symptom did not show improvement, and urinary proteinuria remained at 4+. The patients died at 10 months and 2 months, respectively.

The urinary proteinuria test results in case 6 became negative after antiviral therapy was administered at 3 months of age. The proteinuria levels then increased to 3+ at the 8-months follow-up due to infection and have remained unchanged to date.

Notably, patient 6 had both *NPHS1* mutations (CNS) and COL4A5 gene mutations (Alport syndrome) along with active CMV infection. At onset, the urinary protein/creatinine ratio, urine protein, serum albumin, and CMV IgM were 24.97, 4+, 21 g/L, and 27.7 U/mL, respectively. After ganciclovir therapy, the urine protein/creatinine ratio decreased to 1.63; the urine protein results were negative; serum albumin (without albumin infusion) increased to 30.4 g/L; and no virus was detected in the blood. The CMV IgM was 5.8 U/mL (negative) at 3 months. However, at the 8-months follow-up, the urinary protein increased to 3+ due to infection and has remained stable to date.

The urinary protein results in 2 patients (cases 1 and 5) decreased from 4+ to 2+ after ganciclovir therapy, but these patients were lost to follow-up.

#### Corticosteroid Treatment

In 8 patients with CMV infection, 3 patients (cases 1, 3, and 5) were treated with corticosteroids (prednisone acetate, 1.5–2 mg/kg daily for 1–4 weeks). The dose was gradually tapered and then stopped. The corticosteroids showed no effect on CNS.

#### Other Therapies

One patient (case 1) was administered with cyclosporin 1 mg/kg daily for 2 weeks. One patient (case 3) was administered gamma globulin 1 g/kg. Ten patients (cases 1, 2, 3, 4, 8, 9, 10, 12, 13, and 14) were administered albumin 1 g/kg but not daily albumin infusions in conventional hospitalization.

None of the patients underwent early bilateral nephrectomy, dialysis, and early kidney transplantation.

Outcomes (Table 2).

In all 14 patients, only 2 patients (cases 4 and 7, active CMV infection) showed negative urine protein results at 9 years of age.

One patient (case 6, active CMV infection + *NPHS1* + COL4A5 gene mutations) was followed up with 3+ gross proteinuria and is still being monitored.

One patient (case 11) had 2+ proteinuria and is now 5 years old without edema.

The urinary protein levels in 3 patients fluctuated between 2+ and 4+ (cases 1 and 5 with active CMV infection and case 9 without CMV infection; the parents of these 3 patients refused genetic testing).

The 7 remaining patients died with severe edema between 2 and 10 months of age (mean, 4 months).

## Discussion

CNS is a rare disorder characterized by edema, hypoalbuminemia, gross proteinuria (reaching nephrotic syndrome level), and onset within 3 months after birth. Some patients with CNS have hereditary gene mutations, and others may have underlying diseases, such as CMV infection. From January 1995 to June 2018, 2,583 patients were diagnosed with nephrotic syndrome in the Pediatric Department of Peking University First Hospital; 14 of these patients had confirmed CNS. The worldwide frequency is ~1–3 per 100,000, and it is 1 in 10,000 in Finland. The incidence of children with CNS in China is unclear. Among the children in this hospital, the incidence of children with CNS accounted for 0.54% of children with nephrotic syndrome. In our study, the 14 patients underwent several tests to determine the underlying cause of secondary CNS, such as CMV, HIV, HBV, HCV, herpes simplex virus, syphilis, and toxoplasma infection. Only CMV infection was found.

CMV infection is a common intrauterine infection. In the United States and Europe, the incidence of congenital CMV is 1% (0.5–2%) of live births (5), and the positive rate of maternal CMV IgG in the United States is 65–90% and is even higher in China (90–95%) (6). Furthermore, ~1–4% of CMV sero-negative mothers can become infected during pregnancy, and 30–40% of these infected women will transmit the virus to the fetus.

Secondary maternal CMV infections can also cause fetal transmission. These infections might be reactivated latent infection or re-infection with a new strain in seropositive women. Currently, ~10–30% of women with preconception immunity become re-infected, and 1–3% transmit the virus to the fetus (7, 8).

Both primary and recurrent infections can cause symptoms and long-term neurodevelopmental sequelae in newborns. Based on two recent meta-analyses, 11–12.7% of congenital CMV neonates had symptoms of CMV infection (8). Peri- and post-natal infections were more frequent than congenital infection because the incubation period of CMV was long, and some infections after birth might be asymptomatic for weeks. The clinical manifestations of CMV infection in infants include interstitial pneumonia, lymphadenopathy, and hepatomegaly with hepatitis. Diagnosing infantile CMV infection is difficult. Quantification of viral DNA by using PCR is thought to be sensitive and specific for the diagnosis of congenital CMV infection. The diagnosis of CMV infection in our patients was confirmed based on positive PCR or CMV IgM analysis in serum. CMV infection was found in 8 patients (57.1%).

CMV infection has been frequently reported as an inducing factor for secondary CNS. However, the mechanisms are not well-defined. Two possible mechanisms have been proposed: first, CMV directly damages the kidney tissues; second, CMV triggers an immune response that leads to kidney damage. Renal pathology can be used to distinguish between these two possibilities.

Until 2018, 7 cases of CMV-associated CNS were reported in other countries, and CMV infection was the only secondary finding. In the 7 patients, 5 patients underwent renal biopsy (9–13), and renal tubular cytomegalovirus inclusion bodies were found in 2 patients (9, 10). Of the 7 patients, 5 were administered with antivirus therapy, and proteinuria in 4 patients disappeared, and their clinical conditions improved after ganciclovir treatment (10, 12, 14). However, 1 patient died (13). The remaining 2 patients was not administered antivirus therapy; 1 patient improved after symptom-relieving and supportive treatment (10), and the other showed no response to cyclosporin and corticosteroid therapy (11).

Zhu reported 3 patients with CMV-associated CNS, and none of these patients underwent kidney biopsy. The proteinuria observed in these patients disappeared after 5 days (15) to 2 weeks (16) of antivirus therapy. However, this study did not provide long-term follow-up information or prognosis for these 3 patients.

However, our study showed different results. Among the 14 cases of CNS, 8 were confirmed to have CMV infection. None of these patients showed pulmonary infection, hepatomegaly, anemia, or central nerve system injury. One patient was hospitalized in critically ill condition and died 2 days before antivirus therapy was initiated. Antiviral therapy was effective for two patients who were followed up until 9 years of age, and their urinary protein levels remained negative.

In the other 5 patients, proteinuria in 1 patient improved after antiviral therapy, but the patient also had *NPHS1* gene mutations. During the follow-up period, the urinary protein in this patient increased to 3+ at 13 months of age, and the long-term prognosis has yet to be determined. However, in the other 4 patients, 2 died, and proteinuria in the last 2 patients remained positive before being lost to follow-up.

Renal biopsy in CMV-associated CNS has been reported to reveal diffuse mesangial sclerosis. Obtaining parental consent for renal biopsy for an infant younger than 3 months is difficult in China. Only 1 infant underwent renal biopsy, but the results did not reveal histological characteristics consistent with diffuse mesangial sclerosis. Instead, the histological characteristics resembled mild mesangial proliferative glomerulonephritic disease. The CMV PCR results were negative on the renal biopsy tissue, and no cytomegalic inclusion bodies were observed in the tubular epithelial or glomerular endothelial cells. The genetic analysis of this case revealed *NPHS1* gene mutations. After antiviral therapy, the patient's clinical condition improved, and proteinuria disappeared for ~1 month. However, during the follow-up, the proteinuria level increased gradually. Hence, we recommend renal biopsy for all patients with CNS not only to identify cytomegalic inclusion bodies in the kidney, but also to explore pathological changes and determine whether CNS was caused by CMV infection.

Hereditary CNS includes the Finnish (*NPHS1* gene mutations) and non-Finnish types (mutations in other genes). Moreover, CNS can be caused by mutations in the *NPHS1, NPHS2, WT1, PLCE1*, and *LamB2* genes (17). *NPHS1, NPHS2*, and *PLCE1* mutations can also cause isolated CNS, and *WT1* and *LamB2* mutations can not only cause CNS but also lead to multiple organ damage (1). Previous literature reports that most CNS is caused by genetic mutations. The most common causative gene is the *NPHS1* gene (18, 19). There are few reports on genotype analysis of children with CNS in China. In this study, 8 of the 14 patients underwent genetic testing. Seven patients (87.5%) had gene mutations. All were Finnish type mutations (*NPHS1* gene mutations), which suggested that *NPHS1* mutations are the main cause of hereditary CNS in China. The mean onset age of these 7 patients was 8.4 days after birth, with severe edema or abdominal ascites as the initial symptom, except for 1 patient (case 6) who had proteinuria in a routine urine test. After diagnosis, the patients were administered symptom-relieving and supportive treatment, but the prognoses were poor. Six of the patients died, and only 1 patient (case 6) is still in follow-up.

Case 11 had a family history of kidney disease, but the genetic analysis showed negative results. This could be related to the immature technical method used at the time of analysis (2010). With the improvement of genetic diagnosis technology, we recommend parents of case 11 to undergo a broader set of genetic panel of kidney disease to patients with proteinuria in this family to further clarify the disease-causing gene, but the parents refused.

Currently, most reports in China have shown that *NPHS1* mutations cause CNS. *LAMB2* gene mutations causing CNS have also been reported (20). In this study, all cases that were positive for gene mutation associated with CNS had mutations in the *NPHS1* gene. In the 7 patients with CNS with Finnish-type gene mutations, 3 also had CMV infection. These 3 patients were administered ganciclovir antiviral therapy. Genetic diagnosis is generally accepted as the gold standard for CNS; hence, we assumed that CMV infection observed in the 3 patients was an accompanying condition rather than the cause of CNS. The antiviral therapy might have alleviated the organ damage caused by CMV infection, but the long-term prognosis was poor due to the *NPHS1* mutations. Patients with hereditary CNS may be vulnerable to CMV infection due to the relatively impaired immunity that results from gross immunoglobulin loss during proteinuria in patients with CNS.

Dandge et al. (11) reported a case of *NPHS2* gene mutation CNS associated with CMV infection (21). The ganciclovir antiviral therapy had a weak effect in this case. At 3 years old, the patient underwent renal transplantation. The renal pathology showed glomerular lesions, which progressed to glomerular sclerosis. Generally, hereditary CNS could be confirmed by genetic analysis, and its prognosis is predictably poor. Some of these patients have CMV infection, which could also cause kidney injury. Therefore, these patients had two types of kidney injuries: the gene mutation and CMV infection. After antiviral therapy, the CMV infection could be controlled, and clinical symptoms could be improved with temporarily negative proteinuria (case 6). However, long-term follow-up of kidney function was needed due to the *NPHS1* mutation. Therefore, if a clinically diagnosed patient with CNS has CMV infection, it cannot be directly inferred that the CNS is secondary to the CMV infection. Under the circumstances, genetic testing should be performed. If the genetic mutation is confirmed, the CMV infection is more likely an accompanying condition associated with hereditary CNS.

There have been many improvements in the treatment of CNS. The current therapeutic strategy in CNS proposed by Finnish physicians is schematically as follows: daily albumin infusions in conventional hospitalization, early bilateral nephrectomy, dialysis, and early kidney transplantation.

However, the children in this study did not undergo regular treatment, some patients were administered albumin infusions, but not regular daily infusion, and did not adhere to regular treatment. The possible reasons are: (1) Rarely do Chinese infants undergo kidney transplants because parents lack confidence in improving long-term prognosis through transplantation; (2) Once the disease is diagnosed, in addition to regular antiviral therapy, parents have difficulty in accepting regular angiotensin-converting enzymes inhibitor (ACEI), indomethacin, or anticoagulant therapy; (3) Considering that the prognosis is not ideal, parents often give up treatment, and case 14 was a girl abandoned to the welfare institution.

In summary, after analyzing the clinical characteristics in 14 cases with CNS in our hospital, we reached the following conclusions:

1. The common mutation genotype of hereditary CNS in China is *NPHS1*. The initial clinical manifestation is intractable edema, and the prognosis is poor.
2. Some hereditary CNS is accompanied by CMV infection. After antiviral treatment, proteinuria will decrease even to negative levels in some cases, but long-term follow-up for renal dysfunction is needed due to *NPHS1* mutations.
3. CMV infection may be the underlying cause of CNS, and antiviral therapy may be effective. However, genetic analysis is necessary to exclude hereditary CNS.

## Data Availability

This manuscript contains previously unpublished data. The name of the repository and accession number are not available.

## Ethics Statement

All procedures performed in studies involving human participants were in accordance with the ethical standards of the institutional and/or national research committee and with the 1964 Helsinki declaration and its later amendments or comparable ethical standards.

## Informed Consent

Informed consent was obtained from the parents of all participants included in the study.

## Author Contributions

XH and YC wrote the main manuscript text. HZ provided professional genetics opinion and helped with the related sections in this article. XZ, HX, and YY contributed to the collection of the clinical data. JD and YZ provided the figures and their explanations and assisted with the design of the laboratory tests and gene analysis methods. YJ and JD provided important opinions on CMV infections in neonates and checked the related parts. All authors reviewed the manuscript.

### Conflict of Interest Statement

The authors declare that the research was conducted in the absence of any commercial or financial relationships that could be construed as a potential conflict of interest.
