# Corrigendum: Analysis of 14 Patients With Congenital Nephrotic Syndrome

**DOI:** 10.3389/fped.2019.00471

**Published:** 2019-11-13

**Authors:** Yan Chen, Yanqin Zhang, Fang Wang, Hongwen Zhang, Xuhui Zhong, Huijie Xiao, Yong Yao, Yi Jiang, Jie Ding, Xinlin Hou

**Affiliations:** Department of Pediatrics, Peking University First Hospital, Beijing, China

**Keywords:** congenital nephrotic syndrome, genetic defect, cytomegalovirus infection, clinical manifestation, prognosis

In the original article, there was a mistake in Table 3 as published. In No. 13, the NPHS1 mutation “c.1135C>T, p. Ala379Thr” was incorrect and should be “c.1135C>T, p. Arg379Trp.” Furthermore, in case No. 6, the NPHS1 mutation “c.1339G>A, p. Glu477Lys” should be “c.1339G>A, p. Glu447Lys.” The corrected Table 3 appears below.

**Table 3:** Variant locus analysis in patients with *NPHS1* mutation.

**No**.	**Variation**	**Amino acid change**	**Mutation status**	**Mutation type**
3	c.2663G>A c.3286+5G>A	p. Arg888Lys -	Het Het	Missense
6	c.2396G>T c.1339G>A	p. Gly799Val p. Glu447Lys	Het Het	Missense Missense
8	c.3027C>G c.3478C>T	p. Tyr1009^*^ p. Ary1160^*^	Het Het	Nonsense Nonsense
10	c.1740G>T c.2042G>A	p. Trp580Cys p. Trp681^*^	Het Het	Missense Nonsense
12	c.713-1G>C c.1760T>G	- p. Leu587Arg	Het Het	Missense
13	c.2506+5G>T c.1135C>T	- p. Arg379Trp	Het Het	Missense
14	c.313G>A c.2386G>C	p. Asp105Asn p. Gly796Arg	Het Het	Missense Missense

* Mutations detected in NPHS1 gene.

*Case 14 is an abandoned baby, and no parental verification was performed*.

The authors apologize for this error and state that this does not change the scientific conclusions of the article in any way. The original article has been updated.

